# Psychometric properties of the Chinese version of the Positive Thinking Scale in individuals after hip fracture surgery

**DOI:** 10.1186/s41155-022-00235-x

**Published:** 2022-09-28

**Authors:** Ching-Hui Chien, Yi-Wen Huang

**Affiliations:** 1 College of Nursing, National Taipei University of Nursing and Health Sciences, No.365, Ming-te Road, Peitou District, Taipei City, 112303 Taiwan; 2 Department of Nursing, National Yang Ming Chiao Tung University Hospital, No. 169, Xiaoshe Road, 260006 Yilan City, Yilan County Taiwan

**Keywords:** Psychology, Optimism, Positive thinking, Psychometrics, Hip fracture

## Abstract

Positive thinking is a form of positive cognition and a coping strategy. The Positive Thinking Scale (PTS) is used to measure positive thinking, but the reliability and validity of the PTS-Chinese have yet to be tested. This study aims to examine the psychometric properties of the PTS-Chinese. A total of 154 patients post-hip fracture surgery completed the questionnaire in a hospital in Taiwan between April 2020 and December 2020. The scales in the questionnaire included the PTS, Automatic Thoughts Questionnaire-Positive, Automatic Thoughts Questionnaire-Negative, Barthel Index, and items related to happiness, demographics, and disease treatment. The results of the confirmatory factor analysis and average variance extracted show that the PTS-Chinese version exhibits construct validity. Scores on the PTS-Chinese version are positively related to scores on the Automatic Thoughts Questionnaire-Positive and happiness items and negatively related to scores on the Automatic Thoughts Questionnaire-Negative. This finding indicates that the PTS-Chinese demonstrates concurrent, predictive, and discriminant validity. The scale also presents acceptable reliability and test–retest reliability. Overall, the PTS-Chinese can be used to evaluate and track the positive thinking of patients. Further studies are needed to assess the psychometric properties of the PTS-Chinese in different cultures and ethnic groups.

## Introduction

Positive thinking is a form of positive cognition (Kendall et al., 1989; Kim et al., 2006) and a coping strategy (Bekhet & Zauszniewski, 2013; Kim et al., 2006). Kim et al. define positive thinking as a process in which individuals interact with their environment. During such interaction, individuals exhibit positive cognitive attitudes and coping strategies in pursuit of personal development (Jung et al., 2007; Kim et al., 2006). Researchers have noted that positive thinking comprises three elements: (1) internal cognition guided by individual goals, (2) the belief that things happen for a reason, and (3) positive relationships of an individual with him/herself, God, and family. Individuals with more positive thinking experience higher life satisfaction, better health status, and higher performance (Kirkegaard-Weston, 2005).

Positive psychology focuses on the positive experiences and feelings of individuals, such as well-being, satisfaction, and happiness (Seligman & Csikszentmihalyi, 2000). Studies of the general public (Jung et al., 2007), middle-aged women (Kim & Hur, 2019), and patients with prostate cancer (Chien et al., 2021, 2022) and other types of cancer as well as their caregivers (Hamidou et al., 2018) have shown that positive thinkers have higher well-being, happiness (Chien et al., 2021; Kim & Hur, 2019), life satisfaction (Jung, et al., 2007), mental domain health-related quality of life (Hamidou et al., 2018), and quality of life specific to prostate cancer (Chien et al., 2021). Positive thinking is the mediating factor that affects the relationships between an individual’s health status and economic stress and happiness (Kim & Hur, 2019).

The Positive Thinking Scale (PTS) is used to measure positive thinking, personal satisfaction, and the status of goal pursuit. The preliminary Korean version of the PTS has 25 questions that were developed by reviewing the existing literature and related scales. On the basis of the survey data collected from 145 college students, 18 questions were extracted via exploratory factor analysis. The questions are divided into two dimensions: personal satisfaction (13 questions, which explained 30.55% of the variance; Cronbach’s *α* = 0.88) and goal pursuit (5 questions, which explained 10.24% of the variance; Cronbach’s *α* = 0.72). The authors do not have the information to show the correlation by dimension. The test–retest reliability of the scale is 0.74 at a 4-week interval. Response choices are on a 5-point Likert scale (1 = strongly disagree, 2 = disagree, 3 = neither agree nor disagree, 4 = agree, and 5 = strongly agree). A high score is associated with high positive thinking, high personal satisfaction, and clear goal pursuit (Kim et al., 2006).

One study recruited adults (*n* = 409) for confirmatory factor analysis (CFA) of PTS. The results show that the two-dimensional second-order scale achieves acceptable convergent validity, i.e., root mean square error of approximation (RMSEA) = 0.072 and comparative fit index (CFI) = 0.99. Discriminant validity was supported by the PTS score as being positively correlated with scores of life satisfaction and negatively correlated with scores of stress. The Cronbach’s *α* is 0.85 for the overall scale, 0.82 for the subscale of personal satisfaction, and 0.61 for the subscale of goal pursuit (Kim et al., 2006). Chien et al. (2021) translated the PTS into a Chinese version (PTS-Chinese) by using the back-and-forth translation method. Experts were invited to review the content and surface validity of the scale for patients with prostate cancer. The Cronbach’s *α* of the overall scale was 0.94, the subscale of personal satisfaction was 0.92, and the subscale of goal pursuit was 0.66 (Chien et al., 2022). The reliability and validity of the PTS-Chinese, however, have yet to be tested.

Research shows that the prevalence rate of depression is 23% in persons with a hip fracture (Heidari et al., 2021). Further, patients with depressive symptoms and low positive affect had worse functional recovery (Fredman et al., 2006), whereas high positive affect contributed to better functional recovery (Fredman et al., 2006; Langer et al., 2015). Hence, in the current study, persons with hip fracture surgery were selected as participants to test the psychometric properties of the PTS-Chinese. The aim of the study was to examine the reliability and validity of evidence of the PTS-Chinese in persons post-hip fracture surgery. Based on the literature (Cheung & Wang, 2017; Fornell & Larcker, 1981; Guadagnoli & Velicer, 1988; Koo & Li, 2016; Sun, 2005; Taber, 2018), the hypotheses of this research were as follows: (1) the Cronbach’s *α* and the omega coefficient of the PTS-Chinese version for internal reliability will be at least 0.7; (2) the intraclass correlation coefficient (ICC) of the test–retest reliability of the PTS-Chinese version will be at least 0.9 at a 2-week interval; (3) the factor loading of each item will be at least 0.4, and the CFA model will have acceptable fit and supports the original scale in two dimensions (construct validity); (4) the average variance extracted (AVE) will be at least 0.5 (construct validity); (5) the PTS-Chinese version score will be negatively correlated with the Automatic Thoughts Questionnaire-Negative (discriminant validity); (6) the different age and dependence groups in terms of activities of daily living (Barthel Index) will obtain significantly different scores in the PTS-Chinese version (discriminant validity); (7) the PTS-Chinese version score will be positively correlated with the score of the Automatic Thoughts Questionnaire-Positive (concurrent validity); and (8) the PTS-Chinese version score will be positively correlated with the happiness score (predictive validity) on a researcher-developed scale.

## Methods

### Study design

In the current study, files of orthopedic outpatients in a teaching hospital in Northern Taiwan were used to recruit participants from April 1, 2020, to December 31, 2020. Using a convenience sampling method, individuals with hip fracture surgery were selected as participants.

### Participants

The inclusion criteria were as follows: individuals with hip fracture who had been discharged for more than 3 months post-surgery, older than 20 years, conscious, not handicapped, and able to perform activities of daily living on their own before hip fracture. Individuals with a history of cancer, severe stroke, severe dementia, or mental illness were excluded from the study.

In accordance with the literature, the number of participants should be 3 to 20 times the number of questions in the scale in factor analysis; however, the total number should not be fewer than 100 (Mundfrom et al., 2005). The PTS has 18 questions. After considering the number of available participants and the feasibility of the study, the number was estimated at 153 (1:8.5). The final number of participants was 154. In addition, the number of participants for test–retest reliability was set as 1:1.5 times the number of questions (Park et al., 2018); thus, it was 27.

A total of 160 potential participants met the inclusion criteria. After screening, 154 individuals satisfied the recruitment criteria and were invited to participate in the study, all of whom agreed. All participants completed the first questionnaire, and, of these participants, 50 were invited to answer the second questionnaire; 27 agreed and completed the questionnaire.

The 154 individuals who participated in this study had undergone a hip fracture operation an average of 6.78 ± 3.52 months prior. The average age of the participants was 67.56 years (standard deviation [SD] = 20.30). Married participants accounted for 52.6% of the sample. Participants who received a formal education (completion of elementary school) comprised 60.4%. Finally, participants who practiced Taoism accounted for 63.0% of the sample.

The 27 participants who completed the second questionnaire for test–retest reliability in this study had undergone a hip fracture operation an average of 8.41 ± 3.63 months prior. The average age of the participants was 61.07 years (SD = 22.67). Married participants accounted for 44.5% of the sample. Participants who received a formal education comprised 70.4%. Finally, participants who practiced Taoism accounted for 63.0% (Table 1).

**Table 1:** Demographic characteristics and disease-related information

Variable	Full sample (*n* = 154)	Test–retest sample (*n* = 27)
	*n* (*%*)	*n* (%)
	Mean ± SD [mix–max]	Mean ± SD [mix–max]
Age (years)	67.6 ± 20.3 [20–95]	61.1 ± 22.7 [20–89]
Gender		
Male	58 (37.7)	10 (37.0)
Female	96 (62.3)	17 (63.0)
Marital status		
Single	18 (11.7)	5 (18.5)
Married	81 (52.6)	12 (44.5)
Separated/divorced	10 (6.5)	2 (7.4)
Widowed	45 (29.2)	8 (29.6)
Education		
Cannot read	45 (29.2)	6 (22.2)
Informal education	16 (10.4)	2 (7.4)
Elementary school	35 (22.7)	6 (22.2)
Junior high school	25 (16.2)	3 (11.1)
Senior high school	12 (7.8)	3 (11.1)
College and above	21 (13.7)	7 (26.0)
Religious beliefs		
None	39 (25.3)	7 (25.9)
Buddhism	13 (8.4)	1 (3.7)
Taoism	97 (63.0)	17 (63.0)
Other	5 (3.3)	2 (7.4)
Number of months after surgery	6.8 ± 3.5 [3–12]	8.4 ± 3.6 [3–12]

### Instruments

#### Positive thinking scale

The PTS-Chinese version includes two subscales: personal satisfaction and goal pursuit. This scale has 18 questions. A 5-point scoring method was adopted, and the scores ranged from 18 to 90. A high score is associated with exhibiting high positive thinking, high personal satisfaction, and clear goal pursuit (Chien et al., 2021; Jung et al., 2007; Kim et al., 2006). The original scale in Korean demonstrates validity and reliability (Kim et al., 2006).

### Automatic Thoughts Questionnaire

The Automatic Thoughts Questionnaire has 30 questions. A 5-point scoring method was used to measure the frequency of automatic negative thinking among patients with depression (Hollon & Kendall, 1980). Later, a shorter version of the questionnaire (with 15 or 8 questions) was developed. In the present study, the version with 8 questions was used to measure the frequency of automatic negative thinking among individuals post-hip fracture surgery. The CFA results showed that the 8-question Automatic Thoughts Questionnaire presented a good model fit and can distinguish between cases of depression and non-depression. The scale also exhibited construct and discriminant validity. The Cronbach’s *α* of the scale was 0.85 (Netemeyer et al., 2002). The Chinese version also achieved good reliability and validity (Chen, 2013; Chen & Chou, 2015). In the current study, Cronbach’s *α* was 0.95.

With regard to automatic positive thinking, Kendall et al. (1989) revised the original 30-item Automatic Thoughts Questionnaire. They added 10 questions about automatic positive thinking, forming a 40-item questionnaire. A 5-point scoring method is used to measure the status of automatic positive thinking among patients in the previous week. The automatic positive thinking subscale can be used to distinguish depression cases from other cases of mental illness. The scale exhibited discriminant validity. The Cronbach’s *α* was 0.90 (Kendall et al., 1989). In the current study, the 10-question automatic positive thinking subscale was used. The scale was translated by researchers in Taiwan into a Chinese version, which demonstrated good reliability and validity (Chen, 2013; Chen & Chou, 2015). In the current study, Cronbach’s *α* was 0.97.

#### Barthel index

The Chinese version of the 10-item Barthel index was used to measure the activities of daily living of individuals post-hip fracture surgery. The scale has good reliability, convergent validity, and predictive validity (Hsueh et al., 2001, 2002). Scores range from 0 to 100. Scores from 0 to 90 indicate moderate to complete dependency, scores from 91 to 99 denote mild dependency, and a score of 100 represents complete independence (Mahoney & Barthel, 1965). In this study, Cronbach’s *α* was 0.94.

#### Happiness

The researchers developed an item to measure happiness among patients with a hip fracture. The question was, “How happy were you in the previous week?” Scoring involved the presentation of a 10-cm horizontal line that ran from 0 = “very unhappy” to 100 = “very happy.”

#### Demographic and disease treatment data

Variables include gender, age, marital status, education level, religious beliefs, and number of months after surgery due to hip fracture.

### Procedure

Individuals who met the recruitment criteria were asked to sign a consent form. While the participants were filling in the questionnaire, the researchers provided assistance as needed, including reading questions to the participants and explaining unclear points. After completing the first questionnaire, the participants were asked whether they would be willing to complete the second questionnaire (i.e., PTS). Participants who agreed completed the second questionnaire 2 weeks later.

### Data analysis

AMOS 19.0, IBM SPSS 20.0, and OMEGA for SPSS (Hayes & Coutts, 2020) were used to perform statistical analysis. The inferential statistical methods included independent sample *t* tests, Pearson’s product-moment correlations, CFA with maximum likelihood estimator, ICC, Cronbach’s *α* coefficient, and omega coefficient. The criteria for CFA model fit were as follows: chi-square/degrees of freedom (*df*) ratio < 3, CFI ≥ 0.95, Tucker-Lewis index (TLI) ≥ 0.95, standardized root mean square residual (SRMR) ≤ 0.08, and RMSEA ≤ 0.05 (Hooper et al., 2008; Sun, 2005). When the model fit was poor, the model was adjusted in accordance with the modification index (MI; Hoyle, 1995). Then, the factor loading obtained via CFA was used to calculate the AVE of each subscale and the values above 0.5 were considered s acceptable (Cheung & Wang, 2017; Fornell & Larcker, 1981). The calculation formula for AVE is as follows: (sum of squared standard loadings) divided by (sum of squared standard loadings + sum of observed variable measurement error) (Carter, 2016).

## Results

### Item analysis

The descriptive statistics of each of the items, including the mean, standard deviation, skewness, and kurtosis, for which the data showed a normal distribution, are listed in Table 2. The corrected correlation coefficient between the items and the total score of the personal satisfaction subscale was within the range of 0.63–0.89, and the coefficient between the items and the total score of the goal pursuit subscale was within the range of 0.40–0.85 (Table 3). Based on the total score of the PTS, the upper 27% of scores for the highest group (*n* = 42) and the lower 27% of scores for the lowest group (*n* = 42) were selected for analysis. Independent sample *t* tests were used to examine the differences between the highest and lowest score groups for each item. The results showed statistically significant differences in the scores of each item between the highest and lowest score groups. The *t* value was within the range of 7.78 to 34.75, and all *p* values were less than 0.001.

**Table 2:** Means, standard deviations, skewness, and kurtosis of scale (*n* = 154)

Variable	Mean	SD	Skewness	Kurtosis
Positive Thinking Scale	53.66	14.64	0.32	-1.35
Personal satisfaction subscale	39.01	10.70	0.32	-1.33
1. Interpret positively	2.95	0.96	0.19	-1.15
2. Live positively	3.01	0.92	0.24	-1.26
3. Easily satisfied	3.02	0.97	0.40	-0.87
4. Get along with other people	3.21	0.92	0.07	-0.61
5. Many positive things	3.01	0.96	0.37	-0.93
6. Life smoothly	2.94	0.92	0.48	-0.70
7. A lucky person	2.97	0.89	0.33	-0.83
8. Satisfied with appearance	2.95	0.98	0.36	-0.66
10. As a happy person	3.03	1.00	0.29	-1.01
11. Everything went well	3.04	0.91	0.18	-0.82
13. Satisfied with it	2.99	0.95	0.20	-0.93
14. Manage life with satisfaction	2.88	0.97	0.34	-0.56
15. More good things than bad things	3.01	0.90	-0.01	-0.82
Goal pursuit subscale	14.66	4.11	0.36	-1.15
9. Don’t give up	3.01	0.97	0.42	-0.85
12. Life is a process	3.03	1.01	0.29	-0.91
16. Be successful	2.88	1.05	0.13	-0.75
17. Trying to achieve	2.85	1.00	0.22	-1.16
18. Live with goals	2.89	0.97	0.27	-0.88

**Table 3:** Item analysis, reliability, factor loading, and average variance extracted (*n* = 154)

Variable	Item-total score corrected correlation	Cronbach’s *α* (if item is deleted)	Cronbach’s *α*	Omega coefficient	Confirmatory factor analysis- factor loading	Average variance extracted
Positive Thinking Scale			0.98	0.98		
Personal satisfaction subscale			0.97	0.97		0.75
1	0.89	0.97			0.90	
2	0.89	0.97			0.91	
3	0.88	0.97			0.90	
4	0.82	0.97			0.84	
5	0.87	0.97			0.88	
6	0.88	0.97			0.90	
7	0.86	0.97			0.87	
8	0.86	0.97			0.87	
10	0.89	0.97			0.90	
11	0.87	0.97			0.88	
13	0.86	0.97			0.87	
14	0.63	0.98			0.64	
15	0.84	0.97			0.86	
Goal pursuit subscale			0.88	0.89		0.64
9	0.79	0.84			0.88	
12	0.85	0.82			0.89	
16	0.77	0.84			0.87	
17	0.40	0.92			0.45	
18	0.81	0.83			0.85	
**Variance**					78.7	

### Construct validity and discriminant validity

#### CFA

The CFA model was divided into two dimensions in the second order: personal satisfaction and goal pursuit, in accordance with the structure of the original scale. Factor loading was within the range of 0.45–0.91. Personal satisfaction ranged from 0.64 to 0.91. For goal pursuit, with the exception of item 17, for which the score was 0.45, the items were within the range of 0.85–0.89 (Table 3). This finding indicated that the construct of the scale is acceptable. The original model fit (chi-square = 275.37, *df* = 136, chi-square/*df* = 2.03, *p* < 0.001, CFI = 0.96, TLI = 0.95, RMSEA = 0.082, SRMR = 0.033) is acceptable after adjusting the covariance (e2 and e3, MI = 19.21) in accordance with the MI value (chi-square = 255.26, *df* = 135, chi-square/*df* = 1.89, *p* < 0.001, CFI = 0.96, TLI = 0.96, RMSEA = 0.076, SRMR = 0.032). By further adjusting five covariances according to the MI value (e1 and e2, MI = 9.84; e1 and e3, MI = 14.57; e9 and e16, MI = 8.05; e16 and e17, MI = 8.95; e9 and e18, MI = 5.9), the model fit is more acceptable (chi-square = 199.23, *df* = 130, chi-square/*df* = 1.53, *p* < 0.001, CFI = 0.98, TLI = 0.98, RMSEA = 0.059, SRMR = 0.030). Additional adjustment of the five covariances according to the MI value (e6 and e8, MI = 5.64; e4 and e11, MI = 4.58; e3 and e11, MI = 5.63; e2 and e7, MI = 4.11; e2 and e15, MI = 4.92) resulted in good model fit (chi-square = 173.10, *df* = 125, chi-square/*df* = 1.39, *p* = 0.003, CFI = 0.99, TLI = 0.98, RMSEA = 0.050, SRMR = 0.029).

### AVE

The AVE of the personal satisfaction and goal pursuit subscales of the PTS-Chinese were 0.75 and 0.64, respectively (Table 3), which are higher than 0.5. This finding indicates that the scale achieved construct validity.

#### Correlation and difference of scale scores

The results show that the scores of the PTS-Chinese, personal satisfaction subscale, and goal pursuit subscale are negatively correlated with the scores of the Automatic Thoughts Questionnaire-Negative (*r* =  − 0.68, − 0.68, − 0.42; all *p* values < 0.001). Moreover, the scores of the PTS-Chinese (*t* = 5.74; *p* < 0.001; 95% CI = 8.67, 17.77), personal satisfaction subscale (*t* = 5.50; *p* < 0.001; 95% CI = 5.98, 12.68), and goal pursuit subscale (*t* = 6.08; *p* < 0.001; 95% CI = 2.62, 5.15) of people aged 65 years and above (*n* = 105) are significantly lower than those of people aged below 65 years (*n* = 49). The scores of the PTS-Chinese (*t* = 12.76; *p* < 0.001; 95% CI = 19.17, 26.25), personal satisfaction subscale (*t* = 12.07; *p* < 0.001; 95% CI = 13.62, 18.99), and goal pursuit subscale (*t* = 12.99; *p* < 0.001; 95% CI = 5.43, 7.39) of people who are moderately or severely dependent in terms of their activities of daily living (*n* = 114) are significantly lower than those of people who are mildly dependent or independent in terms of their activities of daily living (*n* = 40). Overall, the data show that the PTS-Chinese exhibits discriminant validity (Table 4).

**Table 4:** Correlation and difference between scale scores (*n* = 154)

Variable	Mean (SD)	Positive thinking	Personal satisfaction	Goal pursuit
		Pearson’s *r*	Pearson’s *r*	Pearson’s *r*
Automatic Thoughts Questionnaire-Positive	19.90 (9.41)	0.81	0.80	0.64
Automatic Thoughts Questionnaire-Negative	17.90 (6.70)	− 0.68	− 0.68	− 0.42
Happiness	49.84 (21.97)	0.92	0.92	0.77
	*n*	Mean (SD)	Mean (SD)	Mean (SD)
Age (years)				
65–95	105	49.46 (12.88)	36.04 (9.52)	13.42 (3.54)
20–64	49	62.67 (14.20)	45.37 (10.39)	17.31 (4.01)
*t* value		5.74	5.50	6.08
Cohen’s *d*		0.97	0.94	1.03
Barthel index (activities of daily living)				
0 to 90 points (moderate to severe dependence)	114	47.76 (11.25)	34.77 (8.28)	12.99 (3.15)
91 to 100 points (mild dependence to independence)	40	70.48 (9.07)	51.08 (6.99)	19.40 (2.50)
*t* value		12.76	12.07	12.99
Cohen’s *d*		2.22	2.13	2.25

All values are significant at *p* < 0.001. *SD* Standard deviation. Cohen’s *d* (mean 2–mean 1)⁄*SD*pooled

### Concurrent validity

The results show that the scores of the PTS-Chinese, the personal satisfaction subscale, and the goal pursuit subscale are positively correlated with the scores of the Automatic Thoughts Questionnaire-Positive (*r* = 0.81, 0.80, 0.64; all *p* values < 0.001). The data indicate that the PTS-Chinese demonstrates concurrent validity (Table 4).

### Predictor validity

The scores of the PTS-Chinese, personal satisfaction subscale, and goal pursuit subscale are significantly correlated with the happiness score (*r* = 0.92, 0.92, 0.77; all *p* values < 0.001). This finding indicates that the PTS-Chinese has predictive validity (Table 4).

### Internal reliability and test–retest reliability

The Cronbach’s *α* values of the PTS-Chinese, personal satisfaction subscale, and goal pursuit subscale are 0.98, 0.97, and 0.88, respectively (*n* = 154), and 0.96, 0.96, and 0.70, respectively (*n* = 27). The omega coefficient of the PTS-Chinese, the personal satisfaction subscale, and the goal pursuit subscale are 0.98, 0.97, and 0.89, respectively (*n* = 154), and 0.96, 0.97, and 0.75, respectively (*n* = 27). The ICC values for test–retest reliability are 0.96, 0.96, and 0.95, respectively (*n* = 27).

## Discussion

This study aims to test the reliability and validity of evidence of the PTS-Chinese used among Chinese individuals post-hip fracture surgery. For the factor loading for the original PTS, item 9 received the lowest score (0.45), while item 17 received a score of 0.64 (Kim et al., 2006). In the current study, the scale dimension is divided into personal satisfaction and goal pursuit during CFA in accordance with the original scale. Item 17 has the lowest factor loading (0.45), and item 9 has a factor loading of 0.88. The factor loadings of the remaining items are higher than 0.5. The model fit of the two dimensions is good and can explain 78.7% of the variance in positive thinking. The literature notes that items with a factor loading of > 0.4 can be still maintained, and the model remains stable (Guadagnoli & Velicer, 1988). Moreover, the AVE values for further testing in the current work are higher than 0.5. The result indicates that the PTS-Chinese achieves construct validity (Cheung & Wang, 2017; Fornell & Larcker, 1981).

In this study, the score of the PTS-Chinese exhibits a significantly negative correlation at low to moderate levels with the score of the Automatic Thoughts Questionnaire-Negative (Hinkle et al., 2003). The positive thinking scores of the population with different dependence levels for their activities of daily living and different ages can be distinguished. Therefore, the PTS-Chinese demonstrates discriminant validity.

In the current study, regardless of the total score on the PTS-Chinese, personal satisfaction subscale, or goal pursuit subscale, the scale has a high positive correlation with the happiness of an individual with a hip fracture during the past week (0.77 to 0.92; Hinkle et al., 2003), indicating that the scale achieves predictive validity, similar to previous research (Kim et al., 2006). Studies on the reliability and validity of the original scale have noted that, if individuals have high positive thinking (*r* = 0.55), high personal satisfaction (*r* = 0.55), and clear goal pursuit (*r* = 0.36), then their life satisfaction is high (Kim et al., 2006).

The test–retest reliability of the 4-week interval original scale is 0.74 (Kim et al., 2006). In the current study, the 2-week interval ICC of the PTS-Chinese is within the range of 0.95–0.96, indicating that the scale has good test–retest reliability (Koo & Li, 2016). In addition, the PTS-Chinese exhibits good internal validity (Taber, 2018). The Cronbach’s *α* value is within the range of 0.70–0.98; this range is similar to the value in the original scale, i.e., 0.72–0.88 (Kim et al., 2006).

The original scale was designed primarily for a healthy population in Korea, and, thus, the reliability and validity of the scale were tested among college students and healthy adults (Kim et al., 2006). In this study, the scores for goal pursuit of individuals post-hip fracture surgery in the community (14.7 points) are lower than those of patients with advanced prostate cancer in Taiwan (17.5 points, mean age 74.8; Chien et al., 2021) and middle-aged women in South Korea (19.3 points; Kim & Hur, 2019). These findings may be related to the effects of an unexpected fracture and changes in activities of daily living when individuals with a hip fracture underwent surgery (Dyer et al., 2016; Langer et al., 2015). In the current study, the mean age of the participants with a hip fracture is 67.6 years. The population above 65 years old accounts for 68.2% of the participants. The goal pursuit of the ailing elderly post-hip fracture surgery may be dissimilar to that of healthy adults or different from that before the fracture occurred. More studies are necessary to verify the reliability and validity of the PTS-Chinese when it is used among individuals from different ethnic groups and cultures.

This study is the first to use the PTS-Chinese for cases of post-operative hip fracture to examine the reliability and validity of this scale. The preliminary results indicate that the PTS-Chinese is a useful tool and demonstrates acceptable reliability and validity. Nevertheless, this study has several limitations. First, the item for measuring happiness is researcher-designed. In the future, a formal scale, such as the Positive and Negative Affect Schedule (Watson et al., 1988) or the State Hope Scale (Snyder et al., 1996), could be used to measure the positive emotions of individuals. Second, although the sample size was determined in the study proposal phase, it is relatively small in terms of the CFA conducted to test the validity of the scale and the 27 individuals used for the test–retest reliability. The original scale was developed on the basis of a healthy adult population. In future research with a large sample size, healthy Chinese adults could be selected as participants to clarify the reliability and validity of the scale. Moreover, this study still cannot provide an exact cut-point for the appropriate degree of positive thinking, a topic that merits future research.

## Conclusions

The PTS-Chinese demonstrates acceptable internal reliability, test–retest reliability, construct validity, concurrent validity, and predictive validity. The scale can be used to evaluate and track the positive thinking of people. More studies are necessary, however, to test the psychometric properties of the PTS-Chinese in different cultures and ethnic groups.

## Data Availability

The data that support the findings of this study are available on request from the corresponding author. The data are not publicly available due to the ethics consideration. The researchers shall maintain the privacy of the participants, and research data should be used only for academic articles.

## Abbreviations

AVE: Average variance extracted
CFA: Confirmatory factor analysis
CFI: Comparative fit index
DF: Degrees of freedom
ICC: Intraclass correlation coefficient
MI: Modification index
PTS: Positive Thinking Scale
RMSEA: Root mean square error of approximation
SD: Standard deviation
SRMR: Standardized root mean square residual
TLI: Tucker-Lewis index
